# Expressions of miR-155 and miR-181 and predictions of their structures and targets in pigs (*Sus scrofa*)

**DOI:** 10.14202/vetworld.2020.1667-1673

**Published:** 2020-08-21

**Authors:** Jirapat Ninsuwon, Pitchaporn Waiyamitra, Atthaporn Roongsitthichai, Win Surachetpong

**Affiliations:** 1 Department of Veterinary Microbiology and Immunology, Faculty of Veterinary Medicine, Kasetsart University, Bangkok, Thailand; 2 Center for Advanced Studies for Agriculture and Food, Institute for Advanced Studies, Kasetsart University, Bangkok, Thailand; 3 Veterinary Clinic Research Unit, Faculty of Veterinary Sciences, Mahasarakham University, Maha Sarakham, Thailand; 4 Office of Academic Affairs, Faculty of Veterinary Sciences, Mahasarakham University, Maha Sarakham, Thailand

**Keywords:** immune system, microRNA, miR-155, miR-181, porcine

## Abstract

**Background and Aim:**

MicroRNAs (miRNAs) are responsible for gene expression control at the post-transcription level in many species. Several miRNAs are required in the regulation of immune responses, such as B-cell differentiation, T-cell receptor signaling pathway, CD4^+^ T cell selection, and so on. Studies on miRNAs have been extensively conducted in humans and mice; however, reports relevant to miRNAs, especially miR-155 and miR-181, in pigs are limited. Consequently, the present study aimed to investigate the structures, target genes, and expressions of miR-155 and miR-181 in various porcine cells and tissues.

**Materials and Methods:**

Five healthy male pigs from a porcine reproductive and respiratory syndrome virus-negative farm were studied. Before slaughter, blood samples were collected for peripheral blood mononuclear cell isolation. After slaughter, samples of spleen, lymph nodes, and forelimb muscles were collected. Both miR-155 and miR-181 were investigated for their structures with RNAfold web server, for their target genes from three online web servers, and for their expressions using polymerase chain reaction (PCR).

**Results:**

The structures of miR-155 and miR-181 contained hairpins with free energies of −35.27 and −35.29 kcal/mole, respectively. Target gene prediction revealed that miR-155 had perfect complementarity with *Socs1* and *Mapk3k14*, while miR-181 had perfect complementarity with *Ddx3x, Nfat5, Foxp1*, and *Mpp5*. PCR showed that both miRNAs were detectable from all investigated cells and tissues. Moreover, the highest expression of both miRNAs was found from the lymph node of the pigs.

**Conclusion:**

Both miR-155 and miR-181 might be involved with the regulation of porcine immune functions as both miRNAs were detected in several cells and tissues of the pigs. In addition, they had very high complementarities with the seed regions of several immune-related genes.

## Introduction

MicroRNAs (miRNAs) are small non-coding RNAs consisting of approximately 19–23 nucleotides and have been characterized as gene-regulating RNAs which manipulate gene expression at the post-transcription level in plants, animals, and humans [1,2]. The previous studies suggested that miRNAs regulate multiple cellular processes, such as cell differentiation [3], cardiac development and regeneration [4], cancer progression [5,6], immune response [7], and host-pathogen interactions [8]. Among biological processes, a great number of miRNAs are associated with the regulation of immune responses, such as miR-155 and miR-181 [9]. In pigs, the miR-155 expression is relevant to the TLR3/TLR4 signaling pathway in response to lipopolysaccharide stimulation [10]. Moreover, miR-155 is significantly upregulated in the lungs of those affected with *Actinobacillus pleuropneumoniae*, associated with an ongoing inflammatory response in necrotic lung tissues [11]. As for miR-181, it plays various important roles in the development of T and B cells [12-14], especially in the control of B-cell differentiation and the selection of CD4^+^ T cell through the inhibition of SHP-2, PTPN22, DUSP5, and DUSP6 genes, which are important for the T-cell receptor signaling pathway [9]. In addition, miR-181 is responsible for controlling infectious pathogens, such as porcine reproductive and respiratory syndrome virus (PRRSV) [15]. It inhibits PRRSV replication by targeting the open reading frame 4 of the virus [16] and downregulates CD163 receptor in blood monocytes and alveolar macrophages, which are considered the main targets for PRRSV [17].

To inhibit protein expression, the nucleotide sequence of the seed region at the 5’ end of miRNAs complementarily binds to the 3’ UTR of the mRNA target gene. It has been shown that the seed region of miRNAs is highly conserved in many vertebrates [18,19]. Therefore, many computer programs, such as TargetScan, miRanda, anamiR, PITA, starBase, miRTar, DIANA-micro T, miRmap, and PicTar, have been developed to predict targets of miRNAs [20-22]. These programs have been applied for the comparison of seed regions by predicting the secondary structure of miRNA and identifying the target mRNA [23,24]. These target prediction programs offer considerable benefits to researchers as they are less time-consuming, more reliable, and highly effective at predicting miRNA targets without extensive laboratory experiments [20,24].

At present, the roles of miR-155 and miR-181 have been studied in humans and some animals. However, comprehensive studies relevant to the characteristics and roles of miR-155 and miR-181 in pigs, such as structures, target genes, and expression levels, have not been largely explored. Consequently, the present study aimed to characterize the structures of miR-155 and miR-188 in porcine tissues using web-based tools and to evaluate the expressions of miR-155 and miR-181 in different porcine cells and tissues.

## Materials and Methods

### Ethical approval

Animal intervention in the present study was approved by the Chulalongkorn University Animal Care and Use Committee (approval numbers 13310019 and 1431086).

### Animals and sample collection

In total, five healthy castrated 3-month-old male pigs from a commercial PRRSV-negative farm were randomly included in the study. Sample collections were conducted in September 2012. The blood collection was individually performed and kept in heparinized containers before slaughter time. Afterward, spleen, lymph nodes, and forelimb muscles were collected, transferred to the laboratory within the same day, and kept in Trizol at −80°C until assay.

### Extraction of peripheral blood mononuclear cells (PBMCs)

The heparinized blood samples were isolated for PBMC within the same day of blood collection. In brief, each blood sample was mixed with phosphate-buffered saline (PBS) in equal proportion and then over-layered on Ficoll HypaquePlus (GE Healthcare, Pittsburg, PA, USA). After that, each sample was centrifuged at 1000× *g* and 22°C for 20 min. The PBMC layer was later collected and incubated on ice with red blood cell lysis buffer to remove excessive red blood cells. Finally, all samples were individually washed twice with PBS and subjected to RNA isolation.

### Primer design

The forward and reverse primers of *Sus scrofa* pre-miR-155 and pre-miR-181 were designed using the PRIMER3 software (version 0.4.0). As for miR-155, miR-155F (GTTAATGCTAATTGTGATAGGGG), and miR-155R (CATCATACCCTGTTAATGCTAAC) were the sequences of forward and reverse primers, respectively. For miR-181, the sequences of forward and reverse primers were miR-181F (CAGTGAACATTCAACGCTGTC) and miR-181R (GCTGATGGTTGGCCATAGG), respectively. The forward and reverse primers of GAPDH were GAPDH_F (AGGTCATCCATGACAACTTCGGCA) and GAPDH_R (AGCACCAGTAGAAGCAG GGATGAT), respectively.

### RNA extraction, cDNA synthesis, and polymerase chain reaction (PCR)

Total RNA extraction was conducted from porcine tissues and cells, including spleen, muscle, lymph node, PBMCs, and PK-15 cell line using TRIzol^®^ reagent (Invitrogen, Waltham, MA, USA), according to the manufacturer’s instructions. In brief, each sample was mixed and homogenized in 1 mL TRIzol solution. RNA from tissues and cells was extracted in chloroform and precipitated with isopropanol. After centrifugation, each RNA pellet was resuspended in molecular grade water and incubated with DNaseI (Promega, Adison, WI, USA) to remove DNA contamination. RNA was quantified using a Nanodrop spectrophotometer 2000 (Thermo Fisher, Waltham, MA, USA). First-strand cDNA synthesis was performed using reverse transcriptase, SuperscriptIII (Thermo Fisher, Waltham, MA, USA), and the OligoDT primer.

The expressions of pri-miR-155 and pri-miR-181 in porcine tissues and cells were performed using PCR with a 20 μL reaction containing 2 μL cDNA template, 0.2 mM of forward and reverse primers, 2 mM MgCl_2_, 0.2 mM of each dNTP, and 1 μL Taq DNA polymerase (ThermoScientific, Waltham, MA, USA); the final volume was adjusted to 20 μL using molecular grade water. The PCR conditions consisted of denaturation at 95°C for 5 min, then 40 cycles of 95°C for 30 s, annealing at 60°C for 30 s, extension at 72°C for 30 s, and a final extension at 72°C for 7 min. Finally, the PCR products were separated in 5% NuSieve^®^ agarose gel (Lonza, Morristown, NJ, USA). For reverse transcription-quantitative PCR (RT-qPCR), the reactions were carried out in a 20 μL reaction mixture containing 0.3 μM of forward and reverse primers, 10 μL of 2× iTaq^™^ universal SYBR green supermix (Biorad, Hercules, CA, USA), 4 μL of cDNA template, and molecular grade water to adjust the final volume. The qPCR cycling conditions were 95°C for 3 min, 40 cycles of 95°C for 10 s, and 60°C for 30 s. Melting curve analysis was performed at the end of the qPCR cycle with temperature from 65°C to 95°C using 0.5°C increments to determine the specific amplification of qPCR reactions. The expressions of pri-miR-155 and pri-miR-181 were normalized to GAPDH, calculated using the ∆∆Cq method for relative gene expression, and reported as the mean value from five pigs.

### Structures and sequence comparisons of miR-155 and miR-181

All porcine miRNA sequences were retrieved from the miRBase database (http://www.mirbase.org). Then, the RNAfold web server (http://rna.tbi.univie.ac.at/cgi-bin/RNAfold.cgi) was used to generate the secondary structures of *S. scrofa* pre-miR-155 and pre-miR-181. TargetScan program (http://www.targetscan.org/) was subsequently used to analyze the sequences of both miR-155 and miR-181 seed regions at the 5’ ends. Finally, the sequences of mature miRNAs were compared using the bioinformatic services of the European Bioinformatics Institute, the European Molecular Biology Laboratory (http://www.ebi.ac.uk).

### MicroRNA target prediction

The predictions of miRNA target for porcine miR-155 and miR-181 were constructed from the algorithms, based on species and type of miRNA, ­generated from three online web server tools: DIANA-microT (http://diana.cslab.ece.ntua.gr/microT/), miRmap (http://mirmap.ezlab.org/), and PicTar (http://pictar.mdc-berlin.de/cgi-bin/PicTar_vertebrate.cgi). When the sequence data of porcine miRNA were not available from the programs, the target prediction was extrapolated from human miRNA sequences as both species shared identical sequence similarity at the seed region. Subsequently, target genes generated from those online tools were mapped with NCBI geneIDs. The biological pathway of target gene function was identified by analyzing and retrieving the target genes using the KEGG PATHWAY Database (http://www.genome.jp/kegg/pathway.html). Thereafter, ten candidate genes with the highest hit scores were selected for further analyses. Finally, the sequences of candidate genes of *S. scrofa* were retrieved from the NCBI database and were compared with the seed region of each mRNA to identify the complementary sequence.

## Results

### Structures and nucleotide sequences of porcine miR-155 and miR-181

**Figure-1:**
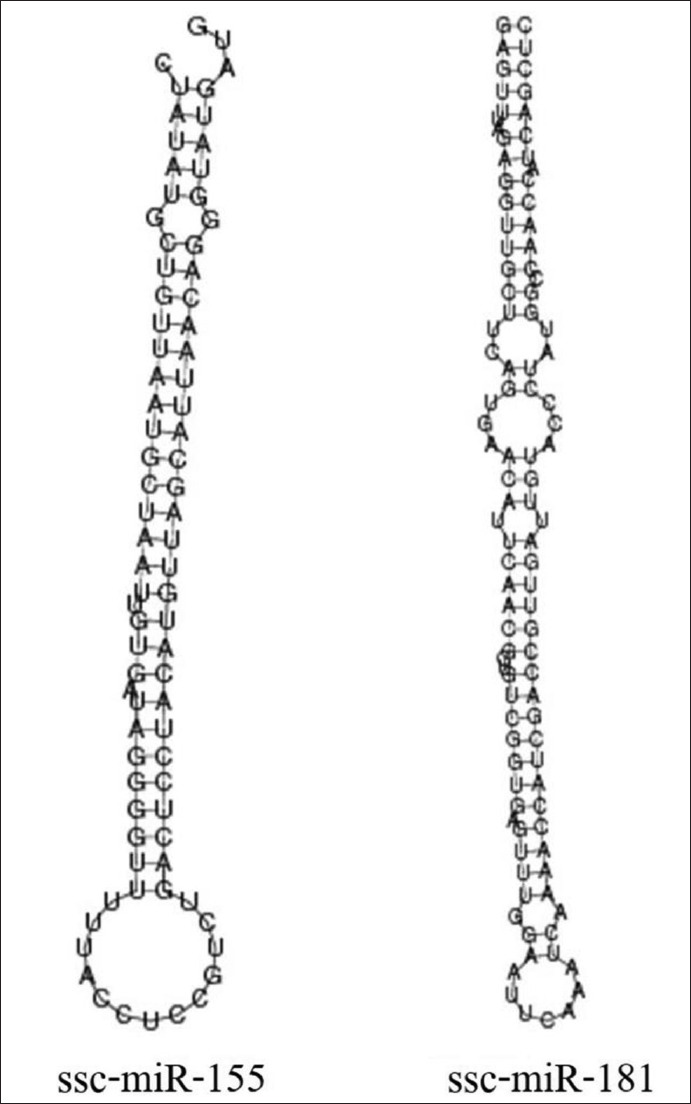
Secondary structures of Sus scrofa miR-155 (ssc-pre-miR-155) and miR-181 (ssc-pre-miR-181) generated by the RNAfold web server.

Based on the analysis of the secondary structures of porcine premature miRNAs (pre-miR-155 and pre-miR-181), it was found that both pre-miR-155 and pre-miR-181 formed hairpin structures with different numbers and sizes of stem-loops (Figure-1). The minimum free energies of pre-miR-155 and pre-miR-181 were −35.27 and −35.29 kcal/mole, respectively. Comparative analysis of both porcine miRNAs with those of other vertebrates: Humans, rats, mice, chickens, and zebrafish, demonstrated that porcine miR-155 shared 91.3-95.7% identity of a nucleotide sequence with other species (Table-1). The nucleotide sequence identity of miR-181 was 95.8% similar to that of other species. Furthermore, an analysis of the 5’ end containing the seed region of miR-155 and miR-181 (UAAUGCU and ACAUUCAA, respectively) showed an identical nucleotide sequence between porcine and other vertebrates (Tables-1 and 2).

**Table-1:** Sequence comparison of porcine miR-155 with other species.

Species	miR-155 (5’-3’)	Similarity to other species (%)	Accession number
*Sus scrofa*	UUAAUGCUAAUUGUGAUAGGGG	100.0	MI0015907
*Homo sapiens*	UUAAUGCUAAUCGUGAUAGGGGU	91.3	MI0000681
*Mus musculus*	UUAAUGCUAAUUGUGAUAGGGGU	95.7	MI0000177
*Rattus norvegicus*	UUAAUGCUAAUUGUGAUAGGGGU	95.7	MI0025509
*Gallus gallus*	UUAAUGCUAAUCGUGAUAGGGG	95.5	MI0001176
*Danio rerio*	UUAAUGCUAAUCGUGAUAGGGG	95.5	MI0002023

**Table-2:** Sequence comparison of porcine miR-181 with other species.

Species	miR-181 (5’-3’)	Similarity to other species (%)	Accession number
*Sus scrofa*	AACAUUCAACGCUGUCGGUGAGUU	100.0	MI0010686
*Homo sapiens*	AACAUUCAACGCUGUCGGUGAGU	95.8	MI0000289
*Mus musculus*	AACAUUCAACGCUGUCGGUGAGU	95.8	MI0000697
*Rattus norvegicus*	AACAUUCAACGCUGUCGGUGAGU	95.8	MI0000953
*Gallus gallus*	AACAUUCAACGCUGUCGGUGAGU	95.8	MI0001218
*Danio rerio*	AACAUUCAACGCUGUCGGUGAGU	95.8	MI0001380

### Porcine miR-155 and miR-181 target prediction

According to the prediction from three online web-based tools, including DIANA-microT, miRmap, and PicTar, target genes related to immune functions of porcine miR-155 consisted of 254, 230, and 199 genes, respectively, whereas those of porcine miR-181 were composed of 509, 577, and 515 genes, respectively. Among those targets, *Bach1*, *Map3k14, and Socs1* had the highest predictive scores for porcine miR-155, while *Ddx3x*, *Foxp1, Mpp5*, *and Nfat5* had the highest predictive scores for porcine miR-181 (Table-3). All of these target genes were associated with porcine immune functions and were predicted by at least two web server tools.

**Table-3:** Prediction of immune-related target genes from three web server tools: DIANA-microT, PicTar, and miRmap.

Type of miR	Gene	Function	Web server tools		

			DIANA-microT	PicTar	miRmap
miR-155	*Bach1*	Transcription regulator protein BACH1 (transcription factor)		✓	✓
miR-155	*Socs1*	Suppressor of cytokine signaling 1 (STAT-induced STAT inhibitor)	✓	✓	
miR-155	*Mapk3k14*	NIK (serine/threonine protein-kinase)	✓	✓	
miR-181	*Ddx3x*	ATP-dependent RNA helicase DDX3X (RNA helicases)	✓		✓
miR-181	*Nfat5*	Nuclear factor of activated T-cells 5 (nuclear factor)	✓		✓
miR-181	*Foxp1*	Forkhead box protein P1(transcription factor)	✓	✓	
miR-181	*Mpp5*	Peripheral membrane-associated guanylate kinase	✓	✓	✓

✓=Target genes validated by the programs. NIK=NF-kappa-B-inducing kinase

**Figure-2:**
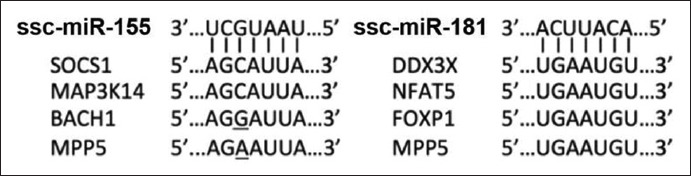
Comparison of seed region of miR-155 and miR-181 with 3’ UTR sequence of target mRNA obtained from the porcine sequence (Source: miRBase database, http://www.mirbase.org).

Due to the pairwise comparison, miR-155 had perfect complementarity with the seed regions of *Mapk3k14* and *Socs1*. The seed regions of *Bach1* and *Mpp5* had only a single mismatch with miR-155 (Figure-2). As for miR-181, it perfectly complemented with the seed regions of *Ddx3x*, *Foxp1, Mpp5*, *and Nfat5* (Figure-2).

### Expression of miR-155 and miR-181 in porcine tissues

**Figure-3:**
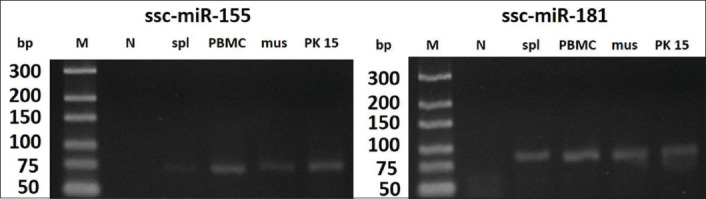
Expressions of pri-miR-155 and pri-miR-181 in porcine tissues. RNAs are extracted from porcine cells and tissues; M=10 bp marker, N=No template control, spl=Spleen, PBMC=Peripheral blood mononuclear cell, mus=Muscle, PK15=PK15 cell line.

**Figure-4:**
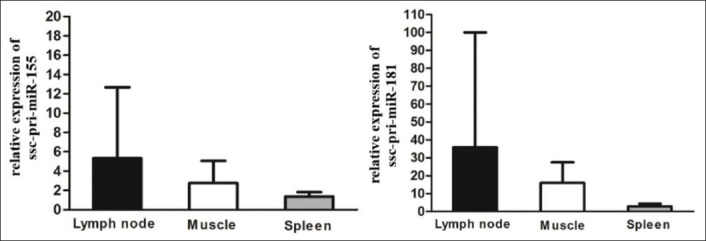
Comparison of ssc-pri-miR-155 and ssc-pri-miR-181 in different porcine tissues. Results are expressed as mean of relative expression of ssc-pri-miR-155 and ssc-pri-miR-181 normalized with GAPDH from five pigs. Data were compared using one-way ANOVA test (p>0.05).

**Figure-5:**
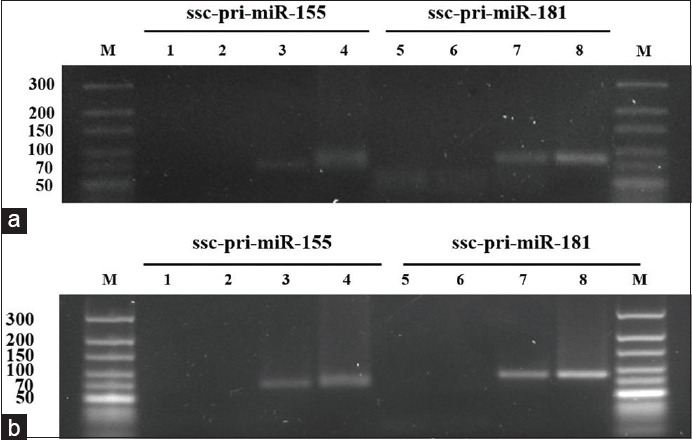
Amplification of ssc-pri-miR-155 and ssc-pri-miR-181 from porcine tissue. (a) No reverse transcription-polymerase chain reaction (RT-PCR) product in no RT-controls M=Molecular marker, Lanes 1, 2, 5, 6=RNA samples from two pigs without RT step (No RT controls), Lanes 3, 7=RNA samples from a pig with RT step, Lanes 4, 8=Plasmid containing porcine miR-155 or miR-181 (positive control). (b) No amplification of PCR product using random hexamer primers; M=Molecular marker, Lanes 1, 2, 5, 6=RNA samples from two pigs using random hexamers in RT step, Lanes 3, 7=RNA samples from a pig RT using oligo (dt) primers, Lanes 4, 8=Plasmid containing porcine miR-155 or miR-181 (positive control).

Based on PCR, it was found that both pri-miR-155 and pri-miR-181 were expressed in PBMCs, spleen, muscle, and PK-15 cell line (Figure-3). Comparison of ssc-pri-miR-155 and ssc-pri-miR-181 in different porcine tissues of five pigs revealed that both miRNAs were expressed in lymph node, muscle, and spleen. Although the expression levels of pri-miR-155 and pri-miR-181 in all three tissues were not statistically significant due to the variation of each pig, we found the highest expression of both miRNAs in the lymph node (Figure-4). No amplification of a specific band in RT-negative control confirmed that there was no DNA contamination in these samples (Figure-5a.). Furthermore, there was no specific amplification of both miR-155 and miR-181 using a random hexamer as a primer for RT reaction (Figure-5b), suggesting that most of the primary miRNAs were in the form with poly A tails.

## Discussion

The present study predicted the molecular structures and nucleotide sequences at the seed regions of porcine miR-155 and miR-181. Moreover, the nucleotide sequences of both miRNAs were highly similar (>90%) to those of various vertebrate species, including humans, rats, mice, chickens, and zebrafish. The previous studies reported that the complementarity of nucleotide sequences between the seed region of miRNA and mRNA targets played important roles in regulating immune functions, especially for mRNA expression [25,26]. It could be implied, according to the high sequence similarity of miRNA between pig and other vertebrates, that porcine miR-155 and miR-181 have similar functionalities involving the modulation of immune functions to those of other vertebrates.

Based on the findings from three web server tools, it was found that the seed region of miR-155 had perfect complementarity of the nucleotide sequence with *Map3k14* and *Soc1*, while that of miR-181 had perfect complementarity of the nucleotide sequence with *Ddx3x*, *Foxp1, Mpp5*, *and Nfat5*. At present, the complete functions of miR-155, miR-181, and their immune-related genes of pigs have not been fully investigated. However, some testimonials relevant to the functions of miR-155 and miR-181 have been reported in other vertebrates. For instance, *Nfat5* regulates the TLR responses in macrophages [27] and has also been revealed as a crucial signaling protein associated with T lymphocyte survival in humans [28]. *Ddx3x* and *Nfat5* have been reported to regulate the immune responses during pathogen infection in humans and mice [29,30]. In fish, the expression of miR-181, as well as miR-29 and miR-125, was low in individuals infected with *Streptococcus agalactiae*. Moreover, *Ddx3x*, including *Atg5*, *Il1a*, and *Lfng*, was controlled by those miRNAs [31]. The functions of porcine miR-155 and miR-181 and target genes could be deduced from the previously reported data of other organisms [32]. Accordingly, miR-155 and miR-181 in pigs might be associated with the regulation of TLR response in macrophages, the survival of T lymphocytes, and the immune regulation affected by pathogens.

Regarding the expressions of miR-155 and miR-181 in porcine cells and tissues, the present study demonstrated that both miRNAs were detected in all examined cells and tissues (Figure-3). Similar to our study, a recent study revealed that miR-181a and miR-181b were highly expressed in alveolar macrophages of two different breeds of pigs during PRRSV infection [33]. This signified that both miRNAs might play some important roles relevant to the regulation of immune functions in those cells and tissues of pigs.

## Conclusion

The expressions of porcine miR-155 and miR-181 were detected in various cells and tissues of pigs. Moreover, molecular structures of both miRNAs were highly similar to those of several vertebrate species. Together with the analyses of their target genes, both miR-155 and miR-181 might be relevant to the regulation of porcine immune function in ways which can be speculated primarily from known functions of their target genes reported in comparable species.

## Authors’ Contributions

JN collected and prepared samples from live and dead pigs, conducted laboratory works, analyzed data using various web server tools, and established the initial version of the manuscript. PW prepared buffers and reagents, together with conducting PCR. AR designed the study, collected and prepared samples from live and dead pigs, interpreted results from web server tools, analyzed data statistically, and finalized the manuscript. WS designed the study, performed PCR, analyzed data from web server tools, and finalized the manuscript.

## Competing Interests

The authors declared that they have no competing interests.

## Publisher’s Note

Veterinary World remains neutral with regard to jurisdictional claims in published institutional affiliation.
